# NFAT Factors Are Dispensable for the Development but Are Critical for the Maintenance of Foxp3^+^ Regulatory T Cells

**DOI:** 10.3390/cells11091397

**Published:** 2022-04-20

**Authors:** Carlotta Barahona de Brito, Amiya Kumar Patra

**Affiliations:** 1 Peninsula School of Medicine, University of Plymouth, Plymouth PL6 8BU, UK; carlotta.barahonadebrito@plymouth.ac.uk; 2 Department of Biology and Biochemistry, University of Bath, Bath BA2 7AX, UK; 3 Department of Molecular Pathology, Institute of Pathology, University of Würzburg, Josef Schneider-Str. 2, 97080 Würzburg, Germany

**Keywords:** thymocytes, NFAT, cyclosporine A, T_reg_ and Foxp3

## Abstract

The transcription factors of the nuclear factor of activated T cell (NFAT) family play a crucial role in multiple aspects of T cell function. It has recently been reported that NFATs play an important role in the suppressive function of CD4^+^CD25^+^Foxp3^+^ regulatory T (T_reg_) cells. In this study, we have investigated the role of NFATs in the thymic development of T_reg_ cells in mice. We show that NFAT factors are dispensable for the development of Foxp3^+^ T_reg_ cells in the thymus but are critical for the maintenance of both the phenotype and survival of T_reg_ cells in the thymus as well as in peripheral lymphoid organs. Specifically, the homeostasis of CD4^+^CD25^+^Foxp3^+^ but not the CD4^+^CD25^−^Foxp3^+^ fraction is severely perturbed when NFAT signaling is blocked, leading to a strongly reduced T_reg_ population. We underscored this intriguing effect of NFAT on CD4^+^CD25^+^Foxp3^+^ T_reg_ cells to the disruption of survival signals provided by interleukin 2 (IL-2). Accordingly, blocking T_reg_ cell death by abolishing the activity of pro-apoptotic Bcl-2 family member Bim, compensated for the survival defects induced due to a lack of NFAT-IL-2-IL-2R signaling. Inhibition of NFAT activity led to a strong reduction in the number of Foxp3^+^ T_reg_ cells; however, it did not influence the level of Foxp3 expression on an individual cell basis. In addition, we show a differential effect of IL-2 and IL-7 signaling on Foxp3^+^ T_reg_ versus CD4^+^CD25^−^ T cell development, again underlining the dispensability of NFAT signaling in the development, but not in the maintenance of Foxp3^+^ T_reg_ cells.

## 1. Introduction

The role of CD4^+^CD25^+^Foxp3^+^ regulatory T (T_reg_) cells in abating the development of autoimmune diseases has been an area of intense study in recent years. Several studies have shown the development of various organ-specific autoimmune disorders in both mouse and human, due to the lack of T_reg_ cells. Foxp3^+^ T_reg_ cells constitute nearly 5% of the mature CD4^+^ single positive thymocytes and up to 15% of the peripheral CD4^+^ T cells. Many recent studies have shown that mouse models having alterations in various signaling pathways bear an increased or decreased population of Foxp3^+^ T_reg_ cells. Although the mechanism of Foxp3^+^ T_reg_ cell-mediated prevention of autoimmunity by suppressing CD4^+^CD25^−^ effector cells is well studied, the signals controlling the development of Foxp3^+^ T_reg_ cells are still not clearly elucidated. It has recently been reported that TGF-β and other signals control the generation of CD4^+^CD25^+^Foxp3^+^ T_reg_ cells in the periphery [1,2,3,4,5] Several recent studies have documented the thymic origin of T_reg_ cells [6,7,8]. Additionally, mice thymectomized at day 3 after birth lack Foxp3^+^ T_reg_ cells in the periphery and develop autoimmune diseases [9,10]. Thus, the thymus plays a major role in the development of Foxp3^+^ T_reg_ cells, although additional mechanisms appear to be involved in their generation in the periphery. Mice deficient in *Tgfb1*, or T-cell-specific knockout for *Tgfbr2*, have a normal thymic development of Foxp3^+^ T_reg_ cells indicating TGF-β signaling is dispensable for the thymic development of these cells [11,12,13]. So far, the precise signals controlling the development of Foxp3^+^ T_reg_ cells in the thymus are not known.

The nuclear factor of activated T cell (NFAT) family transcription factors (TF) play an important role in the development and function of T lymphocytes [14,15,16,17]. Recently, it has been reported that NFATc2 plays a role in the suppressive function of Foxp3^+^ T_reg_ cells by directly interacting with Foxp3. NFATc2-Foxp3 complexes appear to suppress *Il2* and activate *Ctla4* and *Cd25* gene expression in Foxp3^+^ T_reg_ cells [18]. Additionally, NFAT proteins have been reported to play an important role in the functioning of induced-T_reg_ (iT_reg_) cells in the periphery [19]. Thus, it is evident that NFAT proteins play an important role in the functioning of T_reg_ cells. However, it is unknown whether NFAT proteins play any role in the thymic development of natural Foxp3^+^ T_reg_ (nT_reg_) cells. Mice deficient in NFATc2 and NFATc3 show a normal Foxp3^+^ T_reg_ population in the periphery compared to WT mice [20]. Mice deficient in NFATc1 are embryonic lethal [21], and the role of NFATc1 in the development of Foxp3^+^ T_reg_ cells has not been studied. In the current study, we have explored the involvement of NFAT proteins in the thymic development of CD4^+^CD25^+^Foxp3^+^ nT_reg_ cells. We show that NFAT is not necessary for thymic development but is crucial for the maintenance of Foxp3^+^ nT_reg_ cells in the thymus and periphery.

## 2. Materials and Methods

### 2.1. Mice

C57BL/6 WT, calcineurin tg (∆Cam), *Il2*^-/-^, *Il2*^-/-^*Bim*^-/-^, *Nfatc2*^-/-^*Nfatc3*^-/-^, *Jak3*^-/-^, DEREG and *Vav-creNfatc1αA*^fl/fl^ mice were maintained in the animal facility of the Institute for Virology and Immunobiology, University of Wuerzburg, according to institutional guidelines. *Il7r*^-/-^ mice were kindly provided by Dr. Thomas Schueler, Institute of Immunology, Charité, Berlin. All mice were on C57BL/6 background and were used either at day 1 after birth or at 6 weeks of age. For the analysis of aged mice, WT BL/6 mice of over one year of age were used. All mice were used according to institutional guidelines.

### 2.2. Antibodies, Cell Sorting and Flow Cytometry

Anti-CD4 (GK1.5), anti-CD8 (53–6.7), anti-CD25 (PC61 and 7D4), anti-CD3 (145.2C11) and anti-CD28 (37.51) antibodies were all purchased from BD-Pharmingen (Franklin Lakes, NJ, USA). Biotinylated antibodies were revealed with streptavidin-APC. Thymocytes, LN cells or splenocytes were surface stained with anti-CD4, anti-CD8 and anti-CD25 Abs and were analyzed in a FACS Calibure (Becton Dickinson, Heidelberg, Germany). For cell sorting, thymocytes or LN cells were surface stained with anti-CD4, anti-CD8 and anti-CD25 Abs and DN or DP thymocytes, or CD4^+^CD25^−^ and CD4^+^CD25^+^ T cells from LNs were FACS sorted using a FACSVantage cell sorter and FACS DIVA software (Becton Dickinson, Heidelberg, Germany).

### 2.3. Neonatal Thymic Organ Culture (NTOC)

Thymic lobes from one-day-old newborn WT BL/6 mice were cultured on nucleopore membranes (Whatman) in 1 ml cRPMI-1640 medium supplemented with 10% FCS, with PMA (100 ng/mL) plus Ionomycin (100 ng/mL) in presence or absence of CsA (100 ng/mL) or with PMA or Ionomycin alone at 37 °C for 3.5 days. Similarly, *Il2*^-/-^ newborn thymic lobes were cultured in presence or absence of recombinant murine interleukin-7 (IL-7; 20 ng/mL, PeproTech) for 3.5 days. Afterwards, single cell suspensions were surface stained for CD4, CD8 and CD25 and analyzed for the CD4^+^CD25^+^ population gated on CD4^+^ T-cells in FACSCalibure using CellQuest (Becton Dickinson, Heidelberg, Germany) software.

### 2.4. Intracellular Foxp3 and Bcl-2 Staining

Intracellular Foxp3 staining was performed with a rat-anti-mouse Foxp3 staining kit (eBioscience) according to manufacturer’s protocol and as published previously [22]. Briefly, thymocytes or LN cells were first surface stained with anti-CD4, anti-CD8 and anti-CD25 Abs. Surface-stained cells were fixed in 1X Fixperm buffer at room temperature (RT) washed and was stained for intracellular Foxp3 in 1X permeabilization buffer at RT. Afterwards, cells were washed in permeabilization buffer and were analyzed for Foxp3 positive population gated on CD4^+^ T cells using a FACSCalibure and CellQuest software. Intracellular Bcl-2 staining was performed as published previously [16].

### 2.5. Confocal Microscopy

Immunofluorescence staining for confocal microscopy analysis was performed as described previously [16]. Sorted WT CD4^+^CD25^−^ and CD4^+^CD25^+^ T cells from LNs were fixed and stained for NFATc1 (rabbit polyclonal, ImmunoGlobe) and with rat anti-mouse Foxp3 antibodies (eBioscience). Similarly, anti-CD3 (1 μg/mL) plus anti-CD28 (5 μg/mL) antibodies stimulated WT CD4^+^CD25^−^ and CD4^+^CD25^+^ T cells from LNs or sorted DN and DP thymocytes from WT and ∆Cam tg mice were stained with NFATc1 antibodies. In each case, cells were co-stained with DAPI to ascertain nuclear staining. Images were acquired using a Leica TCS SP2 confocal laser-scanning microscope and software.

### 2.6. Cell Death Analysis

Thymic lobes left untreated or treated with CsA (100 ng/mL) at 24 h of NTOC were collected and single cell suspension were prepared. Cells were stained for CD4, CD8 and CD25, and cell death was analyzed by Annexin V staining following manufacturer’s protocol (BD Biosciences).

### 2.7. Statistics

Data are presented as mean ± s.d. Statistical significance was assessed using Student’s *t*-test for comparison between two groups and ANOVA for differences between groups.

## 3. Results

### 3.1. Constitutive Calcineurin/NFAT Activity Does Not Affect the Development of Foxp3^+^ nT_reg_ Cells in the Thymus

We have previously reported that a high and constitutive nuclear NFAT activity blocked T-cell development in calcineurin transgenic (∆Cam) mice at the double negative stage 3 (DN3) [23]. This was due to a severe defect in the formation of preTCR in the DN3 thymocytes, and as a result, only few CD4^+^CD8^+^ double positive (DP) thymocytes were generated in the thymus of these mice. In ∆Cam mice, thymic output was drastically reduced, as is evident from low total thymic cellularity as well as the T-cell populations in the periphery (Supplementary Figure S1a). Compared to WT littermate controls, ∆Cam mice are significantly lymphopenic as the cellularity in the thymus and peripheral lymphoid organs are severely affected (Supplementary Figure S1b). However, in these mice no signs of increased susceptibility to autoimmune diseases or infections were observed until one and half years of age.

**Figure 1:**
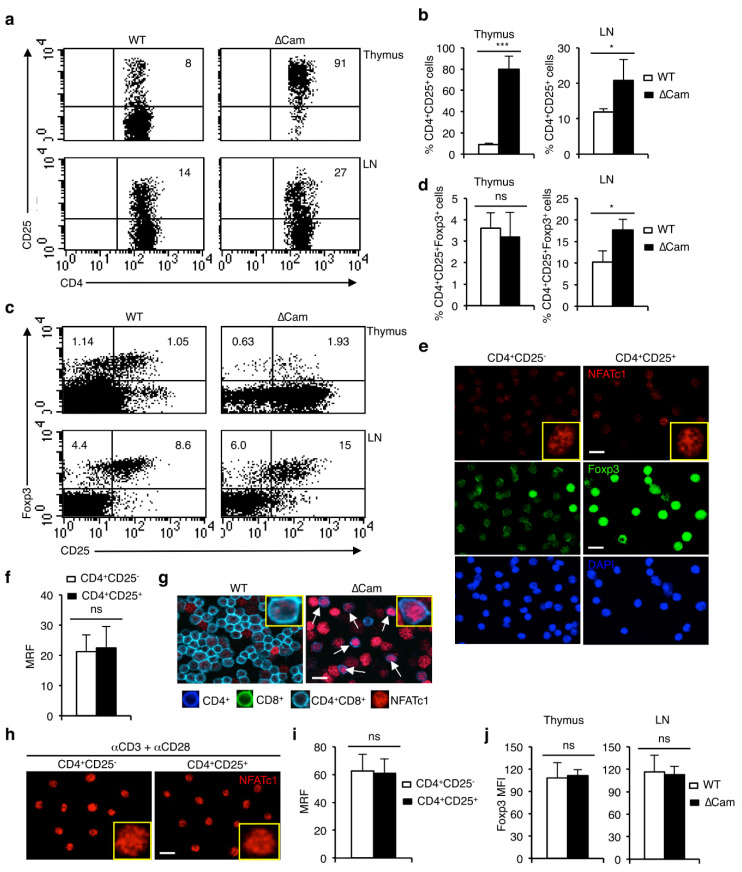
Constitutively active calcineurin/NFAT activity does not affect thymic development of Foxp3^+^ T_reg_ cells. (**a**) Distribution of CD4^+^CD25^+^ cells in the thymus and LNs from WT and ∆Cam tg mice as revealed by flow cytometry. (**b**) Quantification of the frequency of CD4^+^CD25^+^ cells in the thymus and LNs from WT and ∆Cam tg mice (*** *p* < 0.0001 and * *p* = 0.0342; unpaired *t*-test). (**c**) Distribution of CD4^+^CD25^+^Foxp3^+^ cells in the thymus and LNs from WT and ∆Cam tg mice as revealed by flow cytometry. (**d**) Quantification of the frequency of CD4^+^CD25^+^Foxp3^+^ cells in the thymus and LNs from WT and ∆Cam tg mice (ns = not significant and * *p* = 0.0101; unpaired *t*-test). In (**b**,**d**), *n* = 5 each. Numbers inside each dot plot indicate percent respective populations. (**e**) Immunofluorescence staining showing nuclear levels of NFATc1 and Foxp3 in sorted WT CD4^+^CD25^−^ and CD4^+^CD25^+^ populations from LNs. Data represent one of two independent experiments involving pooled LN cells from three mice per experiment. (**f**) Quantification of the mean relative fluorescence (MRF) for NFATc1 levels in CD4^+^CD25^−^ (*n* = 35) and CD4^+^CD25^+^ (*n* = 33) LN cells from WT mice. (**g**) Immunofluorescence analysis of sorted DP thymocytes from WT and ∆Cam tg mice showing nuclear NFATc1 levels. DP cells in ∆Cam tg thymocytes are indicated by arrows. Data represent one of two independent experiments. (**h**) Immunofluorescence staining for NFATc1 in anti-CD3 plus anti-CD28 Abs stimulated CD4^+^CD25^−^ and CD4^+^CD25^+^ T-cells from WT mice. (**i**) Quantification of MRF for NFATc1 levels in anti-CD3 plus anti-CD28 Abs stimulated CD4^+^CD25^−^ (*n* = 24) and CD4^+^CD25^+^ (*n* = 40) T-cells from WT mice. In (**e**,**g**,**h**), nuclear staining was confirmed by DAPI. Scale bar, 10 μm. (**j**) Quantification of the mean fluorescence intensity (MFI) for Foxp3 in thymic and LN Foxp3^+^ cells from WT and ∆Cam tg mice. Data represent one of four independent experiments involving 5 WT and ∆Cam tg mice each. Data in (**b**,**d**,**f**,**i**,**j**) are presented as mean ± s.d.

To study whether the calcineurin/NFAT-mediated defects in T-cell development in ∆Cam mice affect the development of nT_reg_ cells, we analyzed the thymus and LNs from these mice for CD4^+^CD25^+^Foxp3^+^ cells. Although more than 80% of CD4^+^ T-cells in the thymus were CD25^+^, we detected a normal proportion of CD4^+^Foxp3^+^ nT_reg_ population in the thymus of ∆Cam mice, compared to age matched WT mice (Figure 1a–d). However, a significantly increased proportion of both CD4^+^CD25^+^ as well as CD4^+^Foxp3^+^ nT_reg_ populations were observed in the peripheral lymphoid organs of ∆Cam mice (Figure 1a–d). To rule out any interference from the transgenic calcineurin, we analyzed the *Vav-creNfatc1αA*^fl/fl^ mice, in which NFAT activity is enhanced due to the expression of a constitutively active form of NFATc1 in a Cre-dependent manner [17]. In *Vav-creNfatc1αA*^fl/fl^ mice, we observed a similar effect of increased NFAT activity on thymic generation of CD4^+^Foxp3^+^ nT_reg_ cells as in the ∆Cam mice (Supplementary Figure S1c–f). In these mice also the overall T cell development was severely affected [17]. The observations from both these animal models suggest that a high and constitutive NFAT activity does not affect the development of Foxp3^+^ nT_reg_ cells in the thymus, although it exerts a profound negative effect on non-T_reg_ thymocyte development.

Cooperation between Foxp3 and NFAT has been reported to play a role in the suppressor function of T_reg_ cells [18]. However, it is unclear whether NFAT proteins play a role in nT_reg_ cell development. To check whether CD4^+^CD25^−^ effector T cells (T_eff_) and CD4^+^CD25^+^ nT_reg_ cells have any intrinsic difference in NFAT activity, we analyzed nuclear levels of NFATc1 in these cells from WT mice by confocal microscopy. We observed a low but similar levels of nuclear NFATc1 in freshly isolated both CD4^+^CD25^−^ T_eff_ and CD4^+^CD25^+^ nT_reg_ cells (Figure 1e,f), whereas a stark difference in the expression of Foxp3 was observed in these two populations (Figure 1e). Confocal microscopy analysis failed to demonstrate any correlation between NFAT and Foxp3 expression patterns, neither in the nT_reg_ nor in the T_eff_ populations. As the nT_reg_ and T_eff_ cells arise from the DP thymocytes we analyzed the nuclear level of NFATc1 in WT and ∆Cam DP cells. As shown in Figure 1g, ∆Cam DP cells have considerably higher nuclear NFATc1 levels than wild-type DP cells. However, the development of nT_reg_ cell population in the thymus of these mice remained unaffected (Figure 1c,d). Additionally, upon anti-CD3 plus anti-CD28 antibodies stimulation, NFATc1 was predominantly found within the nucleus, to a similar extent in both nT_reg_ and T_eff_ cells (Figure 1h,i), indicating that nT_reg_ cells do not have any inherent defect in NFAT activation. Together with the observations from ∆Cam mice, the confocal microscopy analysis indicates that the level of NFAT expression is not correlated with Foxp3^+^ nT_reg_ cell development. Interestingly, NFAT levels did not influence the levels of Foxp3 expression as both in the ∆Cam and *Vav-creNfatc1αA*^fl/fl^ mice, nT_reg_ cells expressed a similar level of Foxp3 as in WT nT_reg_ cells (Figure 1j and Supplementary Figure S1g).

### 3.2. Deficiency in NFATc2 and NFATc3 Does Not Affect Thymic Development of Foxp3^+^ nT_reg_ Cells

**Figure 2:**
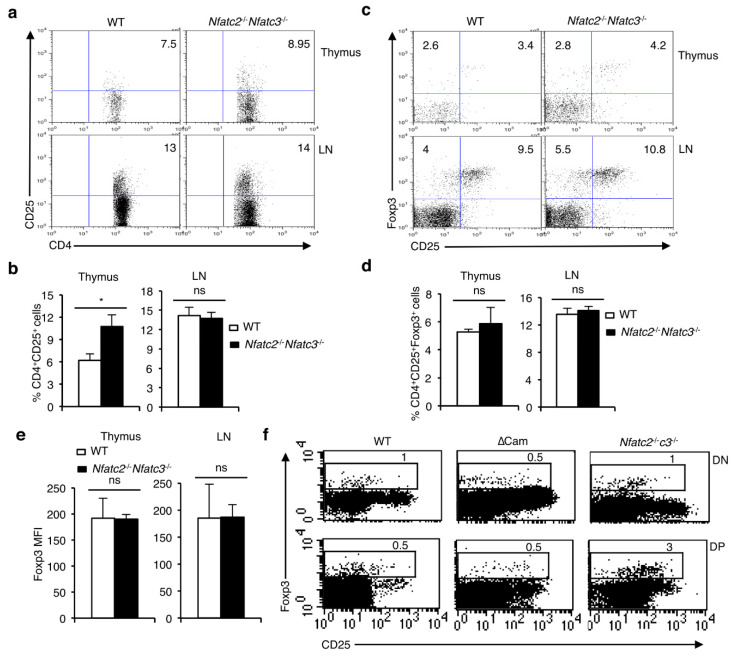
Development of Foxp3^+^ T_reg_ cells is unaffected in *Nfatc2*^-/-^*Nfatc3*^-/-^ mice. (**a**) Distribution of CD4^+^CD25^+^ T cells among CD4^+^ T cell population in the thymus and LN of *Nfatc2*^-/-^*Nfatc3*^-/-^ mice compared to WT littermates. (**b**) Quantification of percent CD4^+^CD25^+^ T cells among CD4^+^ T cell population in the thymus and LN of *Nfatc2*^-/-^*Nfatc3*^-/-^ mice compared to WT controls (*n* = 3 each, * *p* = 0.0120, ns = not significant; unpaired *t*-test). (**c**) Distribution of CD4^+^CD25^+^Foxp3^+^ T_reg_ cells in the thymus and LN of *Nfatc2*^-/-^*Nfatc3*^-/-^ and WT mice gated on CD4^+^ T-cells. (**d**) Quantification of the frequency of CD4^+^CD25^+^Foxp3^+^ T_reg_ cells in the thymus and LNs from WT and *Nfatc2*^-/-^*Nfatc3*^-/-^ mice (*n* = 5 WT and 3 *Nfatc2*^-/-^*Nfatc3*^-/-^ mice; ns = not significant; unpaired *t*-test). (**e**) Quantification of MFI for Foxp3 in thymic and LN Foxp3^+^ cells from WT and *Nfatc2*^-/-^*Nfatc3*^-/-^ mice (ns = not significant; unpaired *t*-test). (**f**) Distribution of Foxp3^+^ cells in DN and DP thymocyte populations from WT, ∆Cam and *Nfatc2*^-/-^*Nfatc3*^-/-^ mice. Data are representative of two independent experiments, and in (**b**,**d**,**e**) are presented as mean ± s.d.

To further clarify the role of NFAT proteins in Foxp3^+^ nT_reg_ cell development, we investigated mice deficient in two of the three predominantly lymphoid specific NFAT family members. Mice deficient in NFATc2 and NFATc3 (*Nfatc2*^-/-^*Nfatc3*^-/-^) do not have any apparent abnormality in thymocyte development whereas the peripheral T-cells from these mice are hyperactive [24]. As shown in Supplementary Figure S2a, the cellularity in the thymus and LNs of *Nfatc2*^-/-^*Nfatc3*^-/-^ mice are comparable to that of littermate control mice. These mice do not have any problem either in the development of thymocytes or in the generation of peripheral CD4^+^ and CD8^+^ T cells (Supplementary Figure S2b). The distribution of CD4^+^CD25^+^ T cells was increased in the thymus of *Nfatc2*^-/-^*Nfatc3*^-/-^ mice, whereas, in the LN it was comparable with that of WT control mice (Figure 2a,b). However, development of Foxp3^+^ nT_reg_ population, neither in the thymus nor in the periphery was affected in the *Nfatc2*^-/-^*Nfatc3*^-/-^ mice (Figure 2c,d). Interestingly, the lack of NFATc2 and NFATc3 did not have any effect on the level of Foxp3 expression in the nT_reg_ cells from *Nfatc2*^-/-^*Nfatc3*^-/-^ mice (Figure 2e), suggesting that Foxp3 expression is independent of NFATc2 and NFATc3 activity. Despite the deficiency of NFATc2 and NFATc3, a distinct Foxp3^+^ population was observed in the DN and DP thymocytes of the *Nfatc2*^-/-^*Nfatc3*^-/-^ mice, as was detected in the WT and ∆Cam mice (Figure 2f). Similar to the CD4^+^CD25^-^Foxp3^+^ population, the Foxp3^+^ cells in DN and DP populations in WT, ∆Cam and *Nfatc2*^-/-^*Nfatc3*^-/-^ mice were found to be CD25 low or negative (Figure 2f). These Foxp3^+^ cell’s presence in the DN and DP thymocytes was confirmed in the DEREG mice [25], where Foxp3 expression could be analyzed by GFP expression (Supplementary Figure S2c,d). This indicates that CD25 expression is not correlated with Foxp3 expression. The CD25^-^Foxp3^+^ cells in the DN population most likely arise from DN4 cells expressing the preTCR, since DN2 and DN3 cells are CD25^+^. This is in consistence with a previous report showing the absence of Foxp3^+^ cells in *Rag1*^-/-^ mice [26]. However, it is unlikely that preTCR signals alone could stimulate the development of this small fraction of Foxp3^+^ cells as the same preTCR signals do not induce Foxp3 expression in the rest of DN4 cells. Similarly, αβ-TCR signals do not seem to induce Foxp3 in the minor fraction of Foxp3^+^ DP cells. Thus, it is unknown which signal(s), alone or in combination with preTCR or αβ-TCR signals, induces Foxp3 expression in thymocytes destined to become nT_reg_ cells. Taken together, the development of Foxp3^+^ nT_reg_ cells appears to be independent of the influence of NFAT proteins, as NFAT overactivity (∆Cam and *Vav-creNfatc1αA*^fl/fl^ mice) or NFATc2 and NFATc3 deficiency (*Nfatc2*^-/-^*Nfatc3*^-/-^) does not affect the generation of these cells.

### 3.3. Cyclosporin A (CsA) Treatment Reduces the Population of Foxp3^+^ T_reg_ Cells Both in the Thymus and in the Periphery

**Figure 3:**
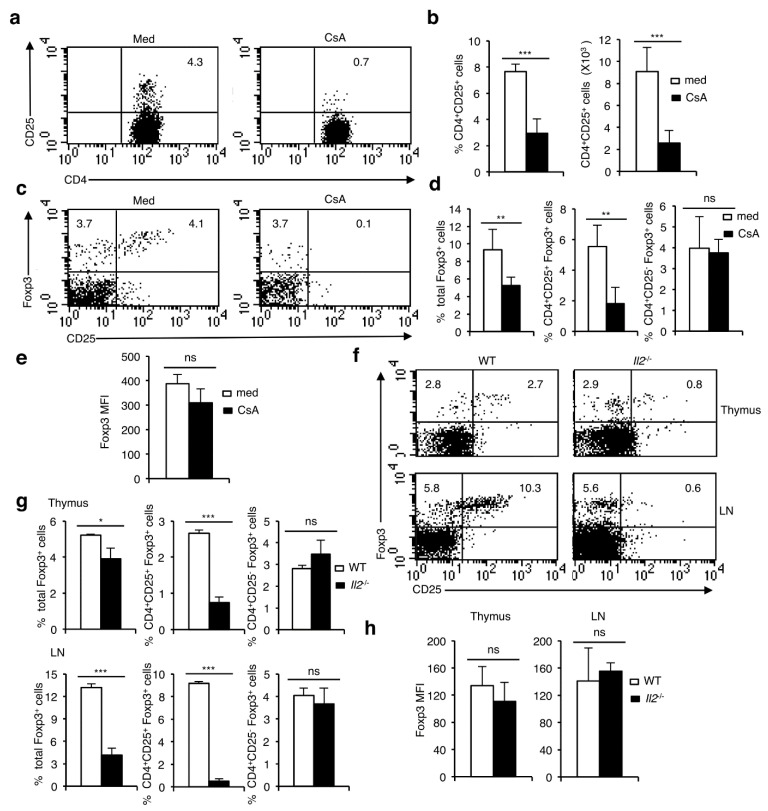
NFAT factors are essential for the maintenance of CD4^+^CD25^+^Foxp3^+^ T_reg_ cells. (**a**) Distribution of CD4^+^CD25^+^ T cells in day 3.5 medium or CsA-treated NTOCs from WT mice. (**b**) Quantification of percent distribution and absolute numbers (*** *p* = 0.0004) of CD4^+^CD25^+^ T cells in day 3.5 NTOCs from WT mice in presence or absence of CsA. (**c**) Flow cytometry showing the distribution of CD25^+^Foxp3^+^ cells gated on CD4^+^ T cells in day 3.5 NTOCs from WT mice in presence or absence of CsA. (**d**) Quantification of percent distribution of total Foxp3^+^ (** *p* = 0.0071), CD25^+^Foxp3^+^ (** *p* = 0.0015) and CD25^-^Foxp3^+^ (ns = not significant) cells gated on CD4^+^ T cells in day 3.5 NTOCs from WT mice in presence or absence of CsA. (**e**) Quantification of MFI for Foxp3 expression levels in Foxp3^+^ cells from day 3.5 NTOCs of WT mice in presence or absence of CsA (ns = not significant). (**f**) Distribution of CD25^-^Foxp3^+^ and CD25^+^Foxp3^+^ cells gated on CD4^+^ T cells in the thymus, and LNs of *Il2*^-/-^ mice compared to WT mice. (**g**) Quantification of percent distribution of total Foxp3^+^ (* *p* = 0.0183; thymus and *** *p* < 0.0001; LN), CD25^+^Foxp3^+^ (*** *p* < 0.0001; both thymus and LN) and CD25^-^Foxp3^+^ (ns = not significant) cells gated on CD4^+^ T cells in the thymus, and LNs of *Il2*^-/-^ mice compared to WT mice. (**h**) Quantification of MFI for Foxp3 expression levels in Foxp3^+^ cells from thymus, and LNs of *Il2*^-/-^ mice compared to WT mice (ns = not significant). Numbers inside each dot plot represent percent respective populations. Data are representative of three independent experiments, and in (**b**,**d**,**e**,**g**,**h**) are presented as mean ± s.d., unpaired *t*-test.

As lymphocytes from *Nfatc2*^-/-^*Nfatc3*^-/-^ mice still express NFATc1, this could compensate for the deficiency of other two members and control nT_reg_ cell development. NFATc1 appears to be the critical NFAT protein that regulate T cell development, as a hematopoietic cells-specific ablation of NFATc1 resulted in a block of T cell development at the DN1 stage [16]. This phenotype of the mice lacking NFATc1 in the hematopoietic cells, ruled out further analysis of T_reg_ cell development in them. However, to study how T_reg_ cell development is affected in complete absence of all NFAT proteins, we analyzed the development of Foxp3^+^ T_reg_ cells in neonatal thymic lobe cultures (NTOC) from newborn wild-type mice cultured in the absence or presence of low doses of CsA. Since CsA blocks NFAT activation by inhibiting calcineurin activity, it should disrupt the effect of all NFAT proteins on Foxp3^+^ T_reg_ cell development. In NTOCs, CsA treatment did not result in any significant alterations in the distribution or number of CD4^+^ T cells compared to the control lobes (Supplementary Figure S3a,b). However, post 3.5 days of CsA treatment, analysis of NTOC shows that CsA strongly downregulated the population of CD4^+^CD25^+^ cells (Figure 3a,b). Due to the reported NFAT-mediated control of CD25 expression in T cells [27], a loss of CD25 expression though was expected upon CsA treatment; however, simultaneously a strong decrease in CD4^+^Foxp3^+^ population was also observed in the CsA-treated lobes (Figure 3c,d). Interestingly, this decrease in Foxp3^+^ cells was restricted to the CD4^+^CD25^+^Foxp3^+^ fraction as the proportion of CD4^+^CD25^-^Foxp3^+^ T_reg_ cells remained unaffected by CsA treatment (Figure 3c,d). Again, blocking NFAT activity by CsA had no effect on the level of Foxp3 expression in the surviving CD4^+^CD25^-^Foxp3^+^ T_reg_ cells (Figure 3e).

To test our assumption that CD25 plays an essential role in the survival (and not in development) of CD4^+^CD25^+^Foxp3^+^ nT_reg_ cells we investigated thymocytes and T cells from mice deficient for IL-2. As shown in Figure 3f,g, and reported by others [28,29], IL-2 deficiency led to a decrease in Foxp3^+^ nT_reg_ population, more strongly in the periphery than in the thymus. Interestingly, similar to the CsA treatment of NTOCs (Figure 3c,d), both in the thymus and in periphery the reduction in Foxp3^+^ nT_reg_ population was mostly due to a specific abolition of CD25^+^Foxp3^+^ cells (Figure 3f,g). On the other hand, the maintenance of CD25^-^Foxp3^+^ nT_reg_ cells (Figure 3f,g), as well as the level of Foxp3 expression (Figure 3h) remained unaffected in the absence of IL-2 signaling.

### 3.4. PMA Signals Negatively Regulate the Development of Foxp3^+^ nT_reg_ Cells in the Thymus

**Figure 4:**
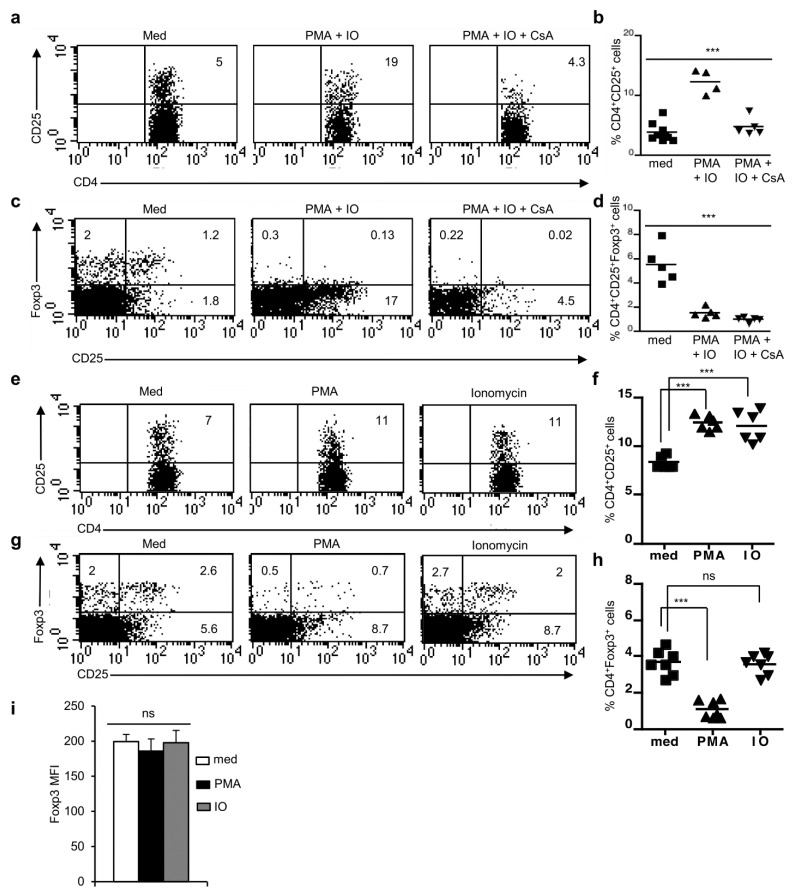
PMA signaling negatively regulates thymic development of Foxp3^+^ T_reg_ cells. (**a**) Distribution of CD4^+^CD25^+^ cells gated on CD4^+^ T cells in day 3.5 NTOCs from WT mice treated with PMA + Ionomycin (IO) in the presence or absence of CsA. (**b**) Quantification of percent distribution of CD4^+^CD25^+^ cells gated on CD4^+^ T cells in day 3.5 NTOCs from WT mice treated with PMA + Ionomycin (IO) in the presence or absence of CsA (*** *p* < 0.0001; one-way ANOVA). (**c**) Distribution of CD25^+^Foxp3^+^ cells gated on CD4^+^ T cells in day 3.5 NTOCs from WT mice treated with PMA+Ionomycin (IO) in the presence or absence of CsA. (**d**) Quantification of percent distribution of CD25^+^Foxp3^+^ cells gated on CD4^+^ T cells in day 3.5 NTOCs from WT mice treated with PMA + Ionomycin (IO) in the presence or absence of CsA (*** *p* < 0.0001; one-way ANOVA). (**e**) Distribution of CD4^+^CD25^+^ cells gated on CD4^+^ T cells in day 3.5 NTOCs from WT mice treated either with PMA or Ionomycin (IO). (**f**) Quantification of percent distribution of CD4^+^CD25^+^ cells gated on CD4^+^ T cells in day 3.5 NTOCs from WT mice treated either with PMA or Ionomycin (IO) (*** *p* < 0.0001; unpaired *t*-test). (**g**) Distribution of CD25^+^Foxp3^+^ cells gated on CD4^+^ T cells in day 3.5 NTOCs from WT mice treated either with PMA or Ionomycin (IO). (**h**) Quantification of percent distribution of CD25^+^Foxp3^+^ cells gated on CD4^+^ T cells in day 3.5 NTOCs from WT mice treated either with PMA or Ionomycin (IO) (*** *p* < 0.0001; ns = not significant; unpaired *t*-test). (**i**) Quantification of MFI for Foxp3 expression levels in Foxp3^+^ cells from day 3.5 NTOCs of WT mice left untreated or treated either with PMA or Ionomycin (IO) (ns = not significant; one-way ANOVA). Data are representative of three independent experiments, and in (**b**,**d**,**f**,**h**,**i**) are presented as mean ± s.d.

To further investigate the role of NFAT proteins in Foxp3^+^ nT_reg_ cell development we treated NTOCs from newborn mice with PMA and ionomycin (IO) in absence or presence of CsA for 3.5 days to induce NFAT activation and to suppress it by CsA. Under these conditions, we did not observe any overt perturbation neither in the distribution of thymic subsets based on CD4 and CD8 stainings nor in the thymic cellularity (Supplementary Figure S4a,b). PMA + IO treatment has been shown to enhance negative selection of self-reactive T cells in the thymus [30]. Analysis of NTOCs showed an increase in the population of CD4^+^CD25^+^ cells in the PMA + IO-treated NTOCs, which was reversed by CsA treatment suggesting the upregulation of NFAT activity and NFAT-mediated CD25 expression by PMA + IO (Figure 4a,b). However, in the PMA + IO-treated NTOCs the development of CD4^+^Foxp3^+^ nT_reg_ cells was severely reduced compared to non-treated NTOCs (Figure 4c,d). This drastic effect of PMA + IO treatment on CD4^+^Foxp3^+^ nT_reg_ cell development was not induced because of high NFAT activity, as CsA treatment of PMA + IO-treated NTOCs did not rescue the development of nT_reg_ cells (Figure 4c,d). This suggests that NFAT proteins are not involved in the PMA + IO-mediated elimination of Foxp3^+^ nT_reg_ cells in the NTOCs.

Next, we wanted to explore which of these two signals (PMA or IO) is responsible for the inhibition of the Foxp3^+^ nTreg cell development in NTOCs. Therefore, we treated NTOCs either with PMA or IO alone and studied the development of Foxp3^+^ nT_reg_ cells 3.5 days later. Under these conditions, neither the thymic subsets distribution, nor cellularity was affected significantly (Supplementary Figure S4c,d). Distribution of CD4^+^CD25^+^ T cells was increased in both PMA- or IO-treated lobes compared to control NTOCs indicating the effectiveness of these treatments (Figure 4e,f). Interestingly, despite a similar increase in CD4^+^CD25^+^ T cells, NTOCs treated with IO alone showed a normal development of Foxp3^+^ nT_reg_ cells, whereas Foxp3^+^ nT_reg_ cell development was severely affected in NTOCs treated with PMA compared to untreated lobes (Figure 4g,h). Both the CD4^+^CD25^+^Foxp3^+^ and CD4^+^CD25^-^Foxp3^+^ populations were severely reduced in PMA-treated NTOCs, whereas development was normal in the control as well as in IO-treated NTOCs. However, under no condition was the level of Foxp3 expression affected in the nT_reg_ cells, and their levels in the few surviving nT_reg_ cells in the PMA-treated lobes were comparable to that of IO-treated or med control cells (Figure 4i). These observations suggest that the thymic development of Foxp3^+^ nT_reg_ cells is independent of NFAT activity and is suppressed by signals generated by PMA.

### 3.5. NFAT Signals Are Not Essential for the Generation of Foxp3^+^ nT_reg_ Cells

**Figure 5:**
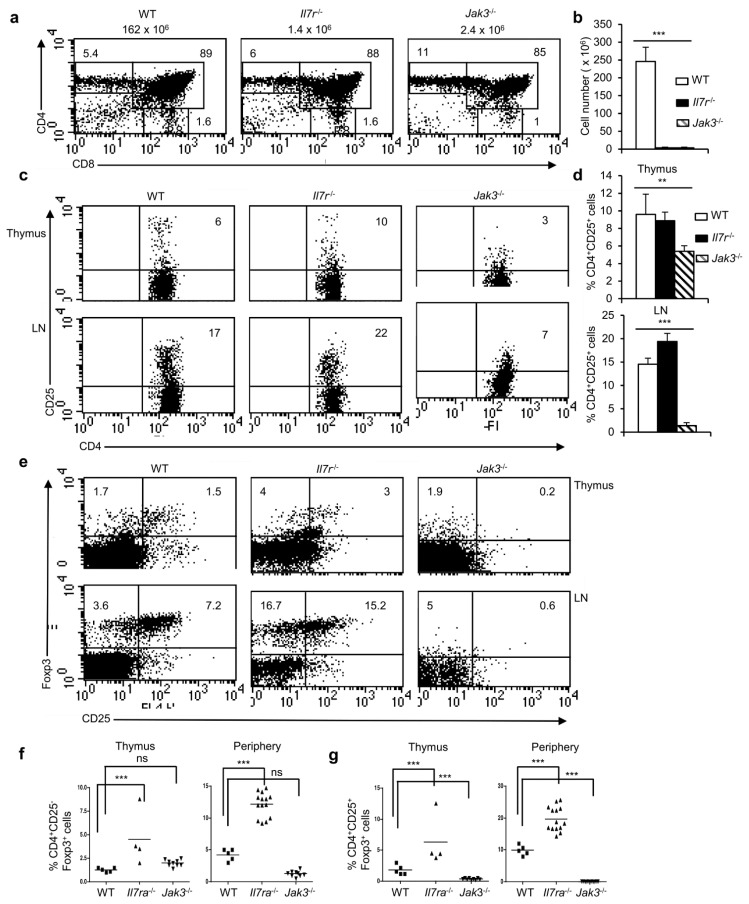
NFAT signaling is dispensable for the generation of Foxp3^+^ T_reg_ cells. (**a**) Flow cytometry showing the distribution of thymocyte populations based on CD4 and CD8 stainings from WT, *Il7r*^-/-^ and *Jak3*^-/-^ mice. Numbers atop each plot represent total cellularity and within each plot represent the percent distribution of respective populations. (**b**) Quanification of total thymic cellularity in WT, *Il7r*^-/-^ and *Jak3*^-/-^ mice (*n* = 7 each, *** *p* < 0.0001; one-way ANOVA). (**c**) Distribution of CD4^+^CD25^+^ cells gated on CD4^+^ T cells in the thymus and LNs from *Il7r*^-/-^ and *Jak3*^-/-^ mice compared to WT controls. (**d**) Quantification of percent CD4^+^CD25^+^ cells gated on CD4^+^ T cells in the thymus and LNs from *Il7r*^-/-^ and *Jak3*^-/-^ mice compared to WT controls (*n* = 6 mice each, ** *p* = 0.0047 and *** *p* < 0.0001; one-way ANOVA). (**e**) Distribution of CD25^+^Foxp3^+^ cells gated on CD4^+^ T cells in the thymus and LNs of indicated mice. (**f**) Quantification of the frequency of CD4^+^CD25^−^Foxp3^+^ cells in the thymus and LNs from *Il7r*^-/-^ and *Jak3*^-/-^ mice compared to WT mice (*** *p* < 0.0001, ns = not significant; unpaired *t*-test). (**g**) Quantification of the frequency of CD4^+^CD25^+^Foxp3^+^ population in the thymus and in LNs from *Il7r*^-/-^ and *Jak3*^-/-^ mice compared to WT mice (*** *p* < 0.0001; unpaired *t*-test). In (**f**,**g**), each dot represents one individual mouse and the horizontal bar represents the mean values. Data are representative of three independent experiments.

Inhibition of NFAT activity in CsA-treated NTOCs led to the disappearance of CD25^+^Foxp3^+^ nT_reg_ cells (Figure 3 and Figure 4) by blocking the expression of CD25 and, therefore, IL-2 signaling via the common γ-chain. Since γ-chain-mediated signals arise not only from IL-2 but also from several other cytokines we wanted to analyze further the signals responsible for the development of Foxp3^+^ nT_reg_ cells in the thymus. Because IL-7 plays a critical role in thymic T cell development as well as in maintenance of both naïve and memory peripheral CD4^+^ and CD8^+^ T cells [31], we analyzed *Il7r*^-/-^ and *Jak3*^-/-^ mice for the development of Foxp3^+^ nT_reg_ cells. T cell development in *Il7r*^-/-^ and *Jak3*^-/-^ mice is strongly impaired and both these mice suffers from severe T cell lymphopenia (Figure 5a,b) [32,33,34,35]. The distribution of CD4^+^CD25^+^ T cells in the thymus and in LNs was similar for both WT and *Il7r*^-/-^ mice, but in case of *Jak3*^-/-^ mice this population was significantly reduced (Figure 5c,d). Intriguingly, in the thymus and periphery of *Il7r*^-/-^ mice, we observed the development of a significantly increased proportion of CD4^+^Foxp3^+^ nT_reg_ cells compared to WT mice (Figure 5e). This result was consistent with the recently reported observation about an enhanced Foxp3^+^ T_reg_ cell development in *Il7*^-/-^ mice [36]. However, both in the thymus and in the periphery of *Jak3*^-/-^ mice the development of Foxp3^+^ nT_reg_ cells was strongly reduced compared to WT control (Figure 5e). Again, the severe reduction in nT_reg_ cells was not due to a general block in the development of Foxp3^+^ nT_reg_ cells but due to the disappearance of CD4^+^CD25^+^Foxp3^+^ T_reg_ population, while the development of CD4^+^CD25^-^Foxp3^+^ T_reg_ cells was normal in *Jak3*^-/-^ mice thymus compared to WT mice (Figure 5f,g). We have reported recently that IL-7-JAK3-mediated NFATc1 activation is critical for thymocyte differentiation and survival at the pTCR-negative stages, and in IL-7R-deficient pTCR-negative cells NFATc1 activation is strongly impaired [16]. Thus, the presence of Foxp3^+^ T_reg_ cells in *Il7r*^-/-^ mice, further suggests that NFAT activity is dispensable for their development.

It has been well established that with age there is a decrease in lymphocyte population both in the thymus and in the periphery leading to a lymphopenic situation similar to that in the IL-7 signaling deficient mice. In the old mice, there was a significant increase in the CD4^+^CD25^+^ population in the spleen compared to 6-week-old mice (Supplementary Figure S5a,b). Interestingly, we also observed a significant increase in CD4^+^Foxp3^+^ nT_reg_ cells in the periphery of WT mice over one year of age, compared to 6-week-old mice (Supplementary Figure S5c,d). This increase in Foxp3^+^ nT_reg_ population was comparable to that in *Il7r*^-/-^ mice (Figure 5) and *Il7*^-/-^ mice, as reported by others [36]. T_reg_ cells have low IL-7Ra expression compared to non-T_reg_ cells [37]. Thus, an inverse correlation seems to exist between the extent of IL-7 signals and the development of Foxp3^+^ nT_reg_ cells, as IL-7 signaling deficiency in vivo in case of *Il7*^-/-^, *Il7r*^-/-^ mice, and in case of aged WT mice, there is a strong increase in Foxp3^+^ nT_reg_ cells. T_reg_ cells independence on IL-7 was evident as in thymic lobe cultures from newborn *Il2*^-/-^ mice, treatment of exogenous IL-7 did not rescue the CD4^+^CD25^+^Foxp3^+^ population from death (Supplementary Figure S5e).

### 3.6. Lack of NFAT Activity Compromises the Survival of nT_reg_ cells

**Figure 6:**
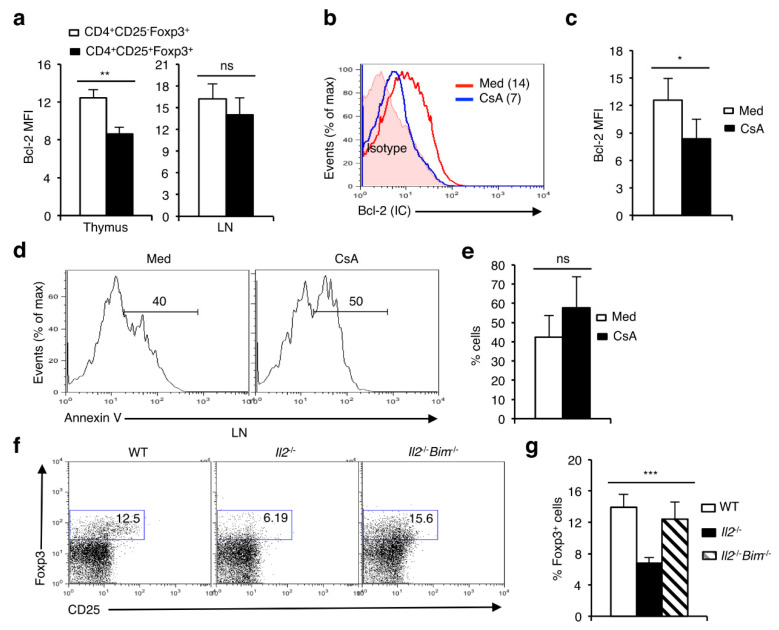
NFAT activity promotes survival and thereby the maintenance of Foxp3^+^ T_reg_ cells. (**a**) Levels of Bcl-2 expression in CD4^+^CD25^−^Foxp3^+^ and CD4^+^CD25^+^Foxp3^+^ cells in the thymus (** *p* = 0.0045; unpaired *t*-test) and in LNs (ns = not significant; unpaired *t*-test) from WT mice. (**b**) Levels of Bcl-2 in CD4^+^Foxp3^+^ cells from 24h NTOC in absence or presence of CsA. Numbers within the plot represent Bcl-2 MFI in respective conditions. (**c**) Quantification of Bcl-2 levels in CD4^+^Foxp3^+^ cells from 24 h NTOC in absence or presence of CsA (* *p* = 0.0401; unpaired *t*-test). (**d**) Cell death in 24 h NTOC in absence or presence of CsA. (**e**) Quantification of cell death in WT 24h NTOC in untreated or CsA-treated lobes (ns = not significant; unpaired *t*-test). (**f**) Restoration of CD4^+^Foxp3^+^ T_reg_ population in *Il2*^-/-^*Bim*^-/-^ mice compared to *Il2*^-/-^ mice. (**g**) Quantification of percent CD4^+^Foxp3^+^ T_reg_ population in *Il2*^-/-^*Bim*^-/-^ mice compared to littermate control mice (*n* = 7 WT, 6 *Il2*^-/-^ and 5 *Il2*^-/-^*Bim*^-/-^ mice, *** *p* < 0.0001; one-way ANOVA). Number inside each dot plot represents % Foxp3^+^ cells. Data are representative of three independent experiments and in (**a**,**c**,**e**,**g**) are presented as mean ± s.d.

NFAT activity has been reported to control both IL-2 and the IL-2Rα (CD25) expression [27,38,39]. Additionally, IL-2 via signaling through its high affinity receptor upregulates Bcl-2 expression and regulates cell survival and function [40,41,42]. To explore whether a lack of NFAT activity affects survival and thereby leads to the development of fewer nTreg cells, we analyzed Bcl-2 levels in these cells. Bcl-2 levels were significantly higher in thymic CD4^+^CD25^−^Foxp3^+^ cells compared to the CD4^+^CD25^+^Foxp3^+^ cells whereas, in the periphery the Bcl-2 levels in both these populations were similar (Figure 6a). Interestingly, blocking NFAT activity by CsA led to a significant decrease in Bcl-2 levels in nTreg cells (Figure 6b,c). This could potentially lead to their death and hence the disappearance of nTreg cells we have observed in the NTOCs (Figure 3 and Figure 4). Accordingly, we have observed enhanced cell death in CsA-treated thymic lobes compared to untreated lobes (Figure 6d,e). We have reported previously that NFATc1 regulates the expression of Bcl-2 in thymocytes and erythrocytes and influences their survival and differentiation [16,43]. Additionally, CsA treatment or shRNA-mediated suppression of NFAT activity resulted in a strong downregulation of Bcl-2 in the thymocytes [16]. Thus, reduced Bcl-2 levels could alter the balance between anti- and pro-apoptotic Bcl-2 family members such as Bim, and thereby influence T_reg_ survival and development. Bim has been reported to be involved in Treg cell death [44,45]. Accordingly, abolition of Bim activity prevented nTreg cell death and restored their development in the IL-2 ko mice (Figure 6f,g). This suggests NFAT is a critical component in regulating multiple parameters related to cell survival, and hence in the maintenance of nT_reg_ cells.

## 4. Discussion

Our observations show that NFAT proteins play an important role in the maintenance and proliferation of CD4^+^CD25^+^Foxp3^+^ T_reg_ cells by controlling CD25 expression and therefore IL-2 signaling, but not in the development of nT_reg_ cells. The continuous up-regulated expression of CD25 on a majority of Foxp3^+^ T_reg_ cells appears to be due to the sustained interaction between Foxp3 and NFAT factors as Foxp3 is always nuclear and has been shown to occupy the regulatory regions of *Cd25* in primary T_reg_ cells [46]. Whereas the activation-induced upregulation of CD25 on conventional CD4^+^ T cells is transient, the constitutive expression of CD25 on T_reg_ cells is quite stable. However, both in activated CD4^+^ T cells as well as in T_reg_ cells, CD25 expression is sensitive to CsA, implicating an involvement of NFAT proteins. The disruption of interaction between Foxp3 and NFAT in CD4^+^CD25^+^Foxp3^+^ T_reg_ cells by CsA treatment leads to the same effect as losing any essential component of the IL-2 signaling cascade. In all these situations, the lack of survival signals will drive the majority of Foxp3^+^ T_reg_ cells into apoptosis leading to a significant reduction in the number of T_reg_ (CD4^+^CD25^−^Foxp3^+^) cells and to a reduced potential to suppress autoimmune diseases.

In our experiments, blockade of NFAT activity by CsA leading to the downregulation of CD25 (IL-2Rα) most likely resulted in the disruption of survival signals provided by IL-2. A similar effect on CD4^+^CD25^+^Foxp3^+^ cells was also observed when LN cells were treated with CsA (data not shown). Due to the strong decrease in Foxp3^+^ T_reg_ population, the net suppressive capacity of the remaining Foxp3^+^ T_reg_ cells might not be sufficient to protect the organism against development of autoimmunity. Our observation could explain the development of T cell-mediated autoimmunity that has been reported to develop in mice treated with repeated doses of CsA [47,48].

The presence of normal numbers of nTreg cells in the *Nfatc2*^-/-^*Nfatc3*^-/-^ mice suggests that most likely the events regulating their maintenance are compensated by NFATc1, which is still present. The lymphocyte hyperproliferation reported in the *Nfatc2*^-/-^*Nfatc3*^-/-^ mice could be due to the functional anomalies in the T_eff_ cells rather than that in the nT_reg_ cells. Due to the constraint of embryonic lethality, NFATc1-deficient mice could not be analyzed for nT_reg_ cell development. However, the observations from CsA-treated NTOCs clearly suggests that nT_reg_ cell maintenance is critically dependent on NFAT activity. The reduced nT_reg_ cell numbers in the *Il2*^-/-^ mice and in all mice lacking any component of the IL-2 signaling pathway indicates that there is a strong dysregulation of cell survival in the absence of IL-2 signals. Here, again the underlying molecular events are regulated by NFAT activity. NFAT has been reported to bind the regulatory elements of *Il2* and *Il2ra* (*Cd25*) and control their expression in T cells [27,38,39]. Downstream of IL-2 signaling, Bcl-2 is an effector molecule that helps T cells survive and discharge their function. NFAT itself has also been shown to regulate Bcl-2 and Bcl-X_L_ in various cell types to influence their survival [23,49,50]. Thus, it is likely that the NFAT-IL-2-IL-2R-Bcl-2 axis is critically essential for T_reg_ cell survival and function, and to keep autoimmunity under control.

The generation of nT_reg_ cells, and their maintenance to keep undue autoimmune reactions under control seem to be differentially regulated by NFAT. Whereas Foxp3 expression levels in nT_reg_ cells were not affected by an increase or decrease in NFAT activity (Figure 1f, Figure 2e, Figure 3e and Figure 4i), the maintenance of the Foxp3^+^ nT_reg_ population was compromised when NFAT activity was abolished (Figure 3 and Figure 4). The presence of Foxp3^+^ nT_reg_ cells in CsA-treated NTOCs, although in a severely reduced number suggests that the generation of nT_reg_ cells is not dependent on NFAT, rather, the reduced nT_reg_ population is due to failure in maintaining the NFAT-IL-2-IL-2R-Bcl-2 signaling axis and the resultant enhanced cell death. This was evident as abolition of Bim activity, prevented the nT_reg_ cell death and restored the full complement of T_reg_ population in the *Il2*^-/-^*Bim*^-/-^ mice (Figure 6f,g). One interesting observation of our study was the unaffected population of CD4^+^CD25^-^Foxp3^+^ cells to NFAT inhibition or IL-2 deficiency (Figure 3). The underlying mechanism for their better survival is unknown. However, we have observed that the thymic CD4^+^CD25^−^Foxp3^+^ cells do express significantly higher levels of Bcl-2 than the CD4^+^CD25^+^Foxp3^+^ cells (Figure 6a).

Several studies have implicated IL-2 signaling to be critical in T_reg_ cell physiology. However, again the generation of Foxp3^+^ cells and in particular the CD4^+^CD25^-^Foxp3^+^ cells was not affected in *Il2*^-/-^ or other IL-2 signaling defective (*Jak*3^-/-^) mice. This suggests that the generation of Foxp3^+^ cells in the thymus as such is not affected in absence of IL-2 signaling. Rather, their maintenance is severely affected, which is more prominent in the periphery than in the thymus. The presence of a distinct Foxp3^+^ population in the CD4^-^CD8^-^ double negative (DN) and CD4^+^CD8^+^ double positive (DP) (Figure 2f and Supplementary Figure S2c,d) thymocytes suggests that the Foxp3^+^ cells are generated much earlier than the differentiating CD4^+^ cells from the DP thymocytes. Similar to the CD4^+^CD25^−^Foxp3^+^ cells, these Foxp3^+^ cells in the DN and DP thymocytes express low levels of CD25. Whether these Foxp3^+^ cells in the DN and DP thymocytes are the precursors for the nT_reg_ cells is an interesting proposition to investigate.

The contrasting observations on the development of CD4^+^CD25^+^Foxp3^+^ T_reg_ cells in the *Il7r*^-/-^ and *Jak3*^-/-^, two severely lymphopenic mice exhibiting a similar defect in thymic development, could be explained by their specific defects in signaling. Whereas in *Il7r*^-/-^ mice, IL-2 signaling was still intact, in *Jak3*^-/-^ mice both IL-7 signals, as well as IL-2/CD25 signals critical for the maintenance of Foxp3^+^ T_reg_ cells were disrupted leading to the reduction in the Foxp3^+^ T_reg_ population. A similar negative effect on Foxp3^+^ T_reg_ population has been reported for mice defective in STAT5 activity, a transcription factor activated by JAK3 [51]. Thus, signaling via the CD25/IL-2α and common γ-chain receptor is essential for the maintenance but not for the development of Foxp3^+^ T_reg_ cells.

We have shown recently that IL-7 signaling-induced NFAT activation is an indispensable feature of T cell development in the thymus [16]. In the absence of NFAT activity, T cell development is severely affected in IL-7 signaling deficient mice. However, the generation of nT_reg_ cells in these mice, that is also at an enhanced level, further suggests that the generation of nT_reg_ cells is independent of NFAT activity. This is also reflected in our analysis of aged animals (Supplementary Figure S5), where IL-7 signaling should be sub-optimal. Altogether, our study reveals an important aspect of NFAT activity in the generation and maintenance of nT_reg_ cells, which could potentially influence multiple clinical conditions such as transplantation, autoimmunity and cancer.

## Supplementary Materials

The following are available online at https://www.mdpi.com/article/10.3390/cells11091397/s1, Figure S1: Enhanced NFAT activity on T cell and nT_reg_ cell development; Figure S2: T cell development in the thymus and LNs of *Nfatc2*^-/-^*Nfatc3*^-/-^ mice; Figure S3: Effects of CsA treament on T cell development in NTOCs; Figure S4: Effects of PMA and IO on thymocytes in absence or presence of CsA in NTOCs; Figure S5: Increase in Foxp3^+^ T_reg_ population with age.
