# Supplementary material for: NFAT Factors Are Dispensable for the Development but Are Critical for the Maintenance of Foxp3+ Regulatory T Cells
Source: Cells. 2022 Apr 20;11(9):1397. doi: 10.3390/cells11091397 (PMC9104130; doi:10.3390/cells11091397)
Supplement: Supplementary file 1 [file cells-11-01397-s001.zip › cells-1653315-supplementary.pdf]

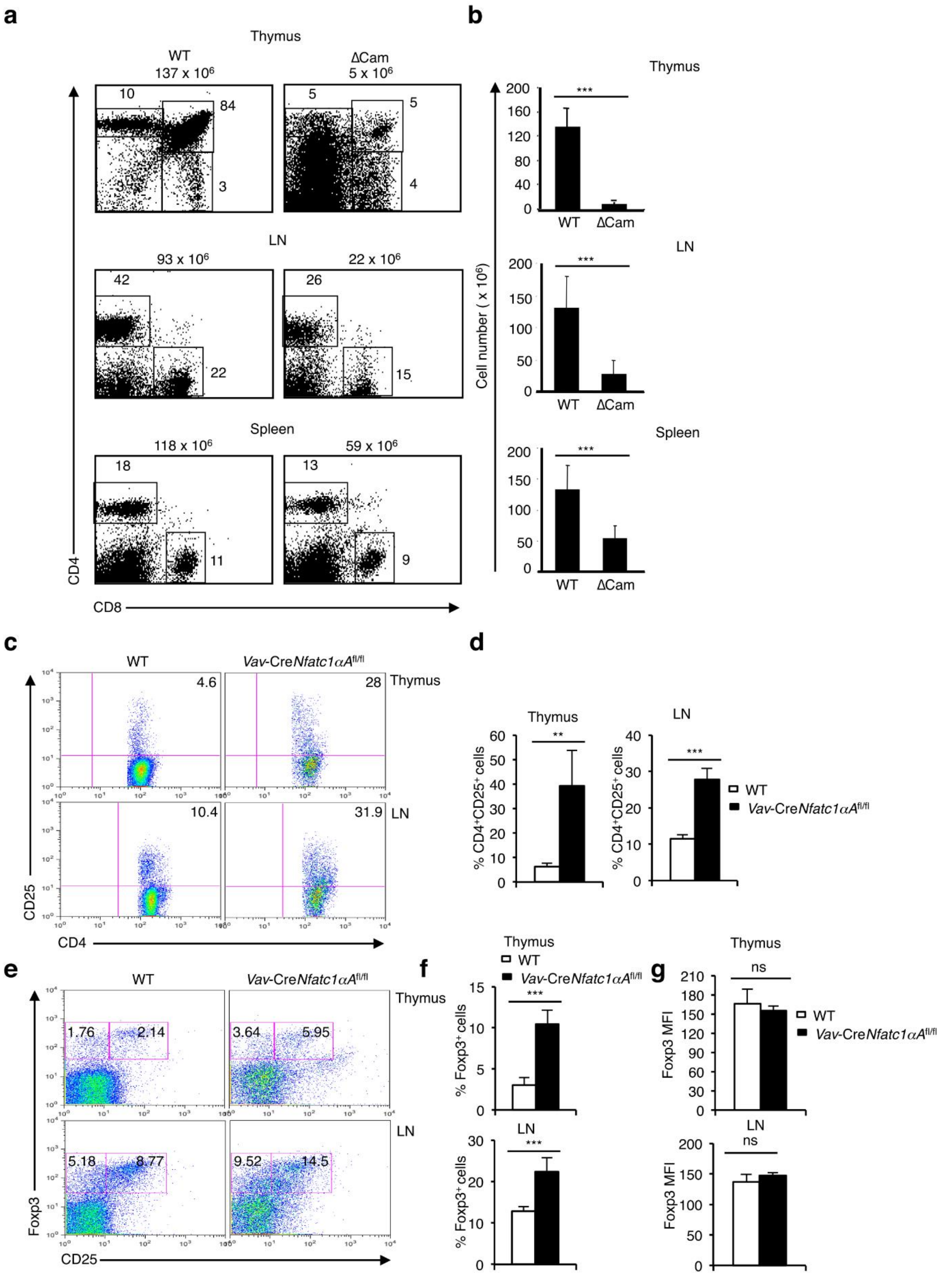

Supplementary Figure S1. Enhanced NFAT activity on T cell and nT$_{reg}$ cell development. (**a**) Distribution of cells based on CD4 and CD8 stainings in thymus, lymph nodes (LN) and spleen of ΔCam tg mice. Number above each dot plot represents the total cellularity. (**b**) Lymphopenia in the ΔCam tg mice. Cellularity in the thymus, LNs and spleen of ΔCam tg

mice compared to WT littermate controls (n = 14 each). (**c**) Flow cytometry profiles showing the distribution of CD4⁺CD25⁺ cells in the thymus and LN from *Vav-creNfatc1αA*$^{fl/fl}$ mice compared to WT controls gated on CD4⁺ cells. (**d**) Quantification of percent CD4⁺CD25⁺ cells in the thymus and LN from *Vav-creNfatc1αA*$^{fl/fl}$ mice compared to WT controls gated on CD4⁺ cells (n = 4 each). (**e**) Distribution of Foxp3⁺ cells among CD4⁺ T cells stained with CD4, CD25 and intracellular Foxp3 in *Vav-creNfatc1αA*$^{fl/fl}$ and WT mice. Numbers inside each dot plot represent percent respective populations. (**f**) Quantification of percent CD4⁺CD25⁺Foxp3⁺ cells in the thymus and LN from *Vav-creNfatc1αA*$^{fl/fl}$ mice compared to WT controls gated on CD4⁺ cells (n = 4 each). (**g**) Quantification of the mean fluorescence intensity (MFI) for Foxp3 in thymic and LN Foxp3⁺ cells from WT and *Vav-creNfatc1αA*$^{fl/fl}$ mice (ns = not significant; unpaired t-test). Data are representative of three independent experiments and in **b** (***$p < 0.0001$; unpaired t-test), **d** (**$p = 0.0038$ and ***$p < 0.0001$; unpaired t-test) and **f** (***$p < 0.0001$; unpaired t-test) are presented as mean ± s.d..

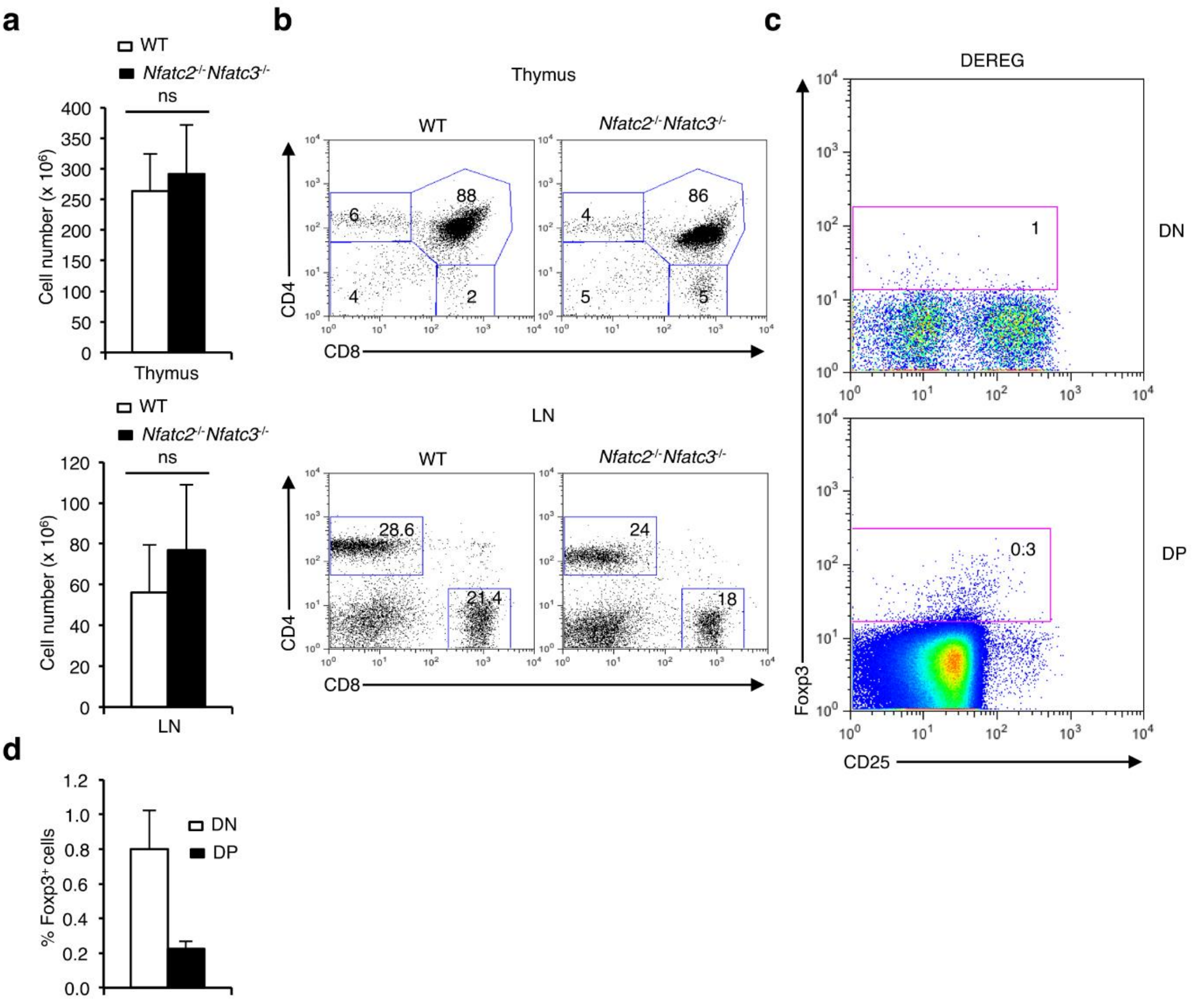

Supplementary Figure S2. T cell development in the thymus and LNs of *Nfatc2*$^{-/-}$*Nfatc3*$^{-/-}$ mice. (**a**) Cellularity in the thymus and LNs of *Nfatc2*$^{-/-}$*Nfatc3*$^{-/-}$ mice compared to WT littermate controls (n = 3 each). (**b**) Subset profile of thymocytes and LN cells from *Nfatc2*$^{-/-}$*Nfatc3*$^{-/-}$ and WT mice stained for CD4 and CD8 surface expression. (**c**) Flow cytometry profiles showing the distribution of Foxp3⁺ cells within DN and DP thymocytes in DEREG mice. Total thymocytes were surface stained for CD4, CD8 and CD25 for the analysis. (**d**) Quantification of percent Foxp3⁺ cells within DN and DP thymocytes in DEREG mice (n = 7). Numbers within each plot give the percentage of individual subsets. Data are representative of three independent experiments and in **a** (ns = not significant; unpaired t-test) and **b** are presented as mean ± s.d..

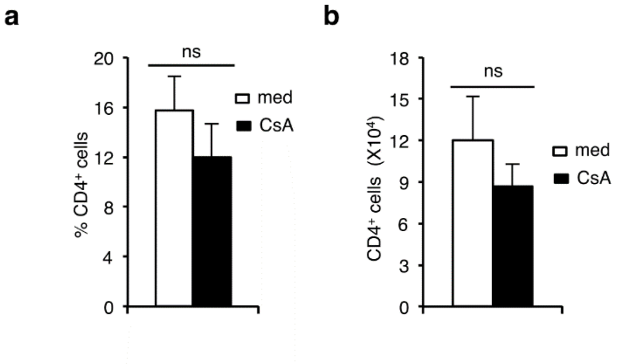

Supplementary Figure S3. Effects of CsA treament on T cell development in NTOCs. (**a**) Quantification of % CD4+ T cells in the untreated or CsA treated WT thymic lobes from NTOCs. (**b**) Quantification of absolute numbers of CD4+ T cells in CsA treated WT thymic lobes compared to untreated lobes. Data are representative of four independent experiments (ns = not significant; unpaired *t*-test) and are presented as mean ± s.d..

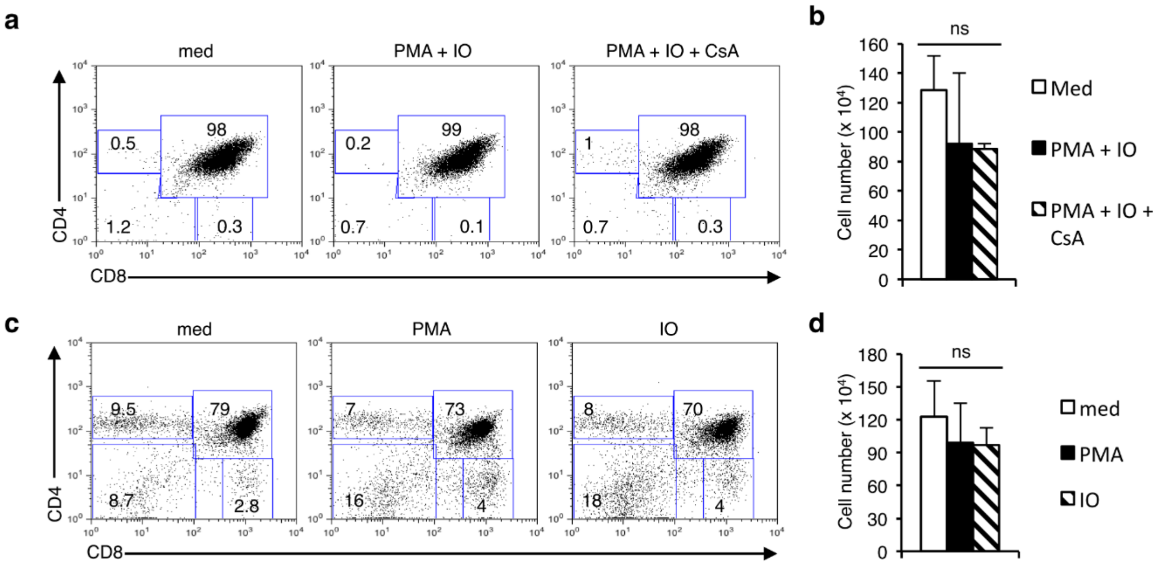

Supplementary Figure S4. Effects of PMA and IO on thymocytes in absence or presence of CsA in NTOCs. (**a**) Flow cytometry profiles showing the distribution of thymic subsets based on CD4 and CD8 stainings in untreated or PMA plus IO treated WT NTOCs in absence or presence of CsA. (**b**) Quantification of total thymocyte numbers from WT thymic lobes left untreated or treated with PMA + IO or PMA + IO + CsA. (**c**) Distribution of thymocyte subsets based on surface CD4 and CD8 stainings from untreated and PMA or IO treated WT thymic lobes. Numbers within each dot plot represent percent respective populations. (**d**) Quantification of total thymocyte numbers from WT thymic lobes left untreated or treated with PMA or IO. Data are representative of four independent experiments and in **b** and **d** (ns = not significant; one-way ANOVA) are presented as mean ± s.d..

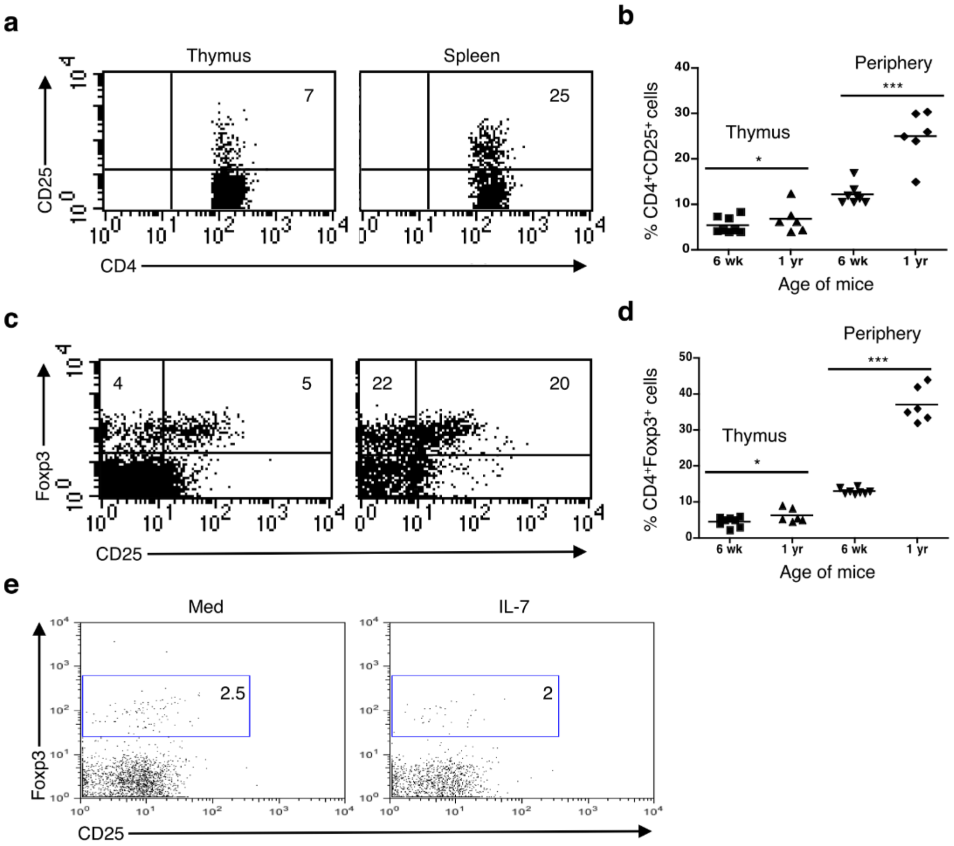

Supplementary Figure S5. Increase in Foxp3⁺ T_{reg} population with age. (a) Flow cytometry analysis shows the frequency of $CD4^+CD25^+$ population in the thymus and spleen of 1yr old WT mice. (**b**) Quantification of the frequency of $CD4^+CD25^+$ cells in young (6 wks) and old (1yr) WT mice. (**c**) Distribution of $Foxp3^+$ population in the thymus and spleen of 1yr old WT mice. Cells were stained for CD4, CD8, CD25 and intracellular Foxp3. (**d**) Quantification of the frequency of $Foxp3^+$ cells in young (6 wks) and old (1yr) WT mice. (**e**) Flow cytometry profiles showing the distribution of $CD4^+CD25^+Foxp3^+$ cells gated on $CD4^+$ cells from NTOCs of *Il2*$^{-/-}$ mice in presence or absence of IL-7 (20 ng/ml) for 3.5 days. Numbers within each plot represent the percent respective populations. Data are representative of three independent experiments (**a**-**d**) and two experiments (**e**), and in **b** (*p = 0.0340 and ***p < 0.0001; unpaired t-test) and in **d** (*p = 0.0260 and ***p < 0.0001; unpaired t-test) are presented as mean ± s.d..
